# ArsenicSkinImageBD: A comprehensive image dataset to classify affected and healthy skin of arsenic-affected people

**DOI:** 10.1016/j.dib.2023.110016

**Published:** 2023-12-28

**Authors:** Ismot Ara Emu, Nishat Tasnim Niloy, Bhuyan Md Anowarul Karim, Anindya Chowdhury, Fatema Tuj Johora, Mahamudul Hasan, Tanni Mittra, Mohammad Rifat Ahmmad Rashid, Taskeed Jabid, Maheen Islam, Md. Sawkat Ali

**Affiliations:** Department of Computer Science and Engineering, East West University, Aftabnagar, Dhaka, Bangladesh

**Keywords:** Arsenic disease dataset, Dermatology, Image classification, Artificial intelligence model, Skin image, Computer vision

## Abstract

Compared to other popular research domains, dermatology got less attention among machine learning researchers. One of the main concerns for this problem is an inadequate dataset since collecting samples from the human body is very sensitive. In recent years, arsenic has emerged as a significant issue for dermatologists. Arsenic is a highly toxic substance found in the earth's crust whose small amounts can be very injurious to the human body. People who are exposed to arsenic for a long time through water and food can get cancer and skin lesions. With a view to contributing to this aspect, this dataset has been organized with the help of which the researchers can understand the impact of this contamination and design a solution using artificial intelligence. To the best of our knowledge, this is the first standard, easy-to-use, and open dataset of arsenic diseases. The images were collected from four places in Bangladesh, under the Department of Public Health Engineering, Chapainawabganj, where they are working on arsenic contamination. The dataset has 8892 skin images, with half of them showing people with arsenic effects and the other half showing mixed skin images that are not affected by arsenic. This makes the dataset useful for treating people with arsenic-related conditions. Eventually, this dataset can attract the attention of not only the machine learning researchers, but also scientists, doctors, and other professionals in the associated research field.

Specifications Table

**Table:** 

Subject	Dermatology
Specific subject area	Image classification, Image processing, Computer vision
Data format	Raw and processed
Type of data	Skin image classification using machine learning which is affected by arsenic
Data collection	In this research, it is considered that arsenicosis i.e., arsenic poisoning which affects human skin is one of the most significant symptoms. To gather data from different areas of Bangladesh, four villages were chosen based on the number of people affected. These villages were identified as affected areas by the Public Health Department in Chapainawabganj. 1482 pictures of both affected and healthy samples were captured for the research.
Data source location	1. Village: Betbaria, Ward:08, Union: Balidanga, Sadar, Chapainawabganj (Latitude: 24° 36′ 56.3′', longitude: 88° 16′ 1.60′') 2. Village: Balubagan, Ward:08, Union: Maharajpur, Sadar, Chapainawabganj (Latitude: 24° 35′ 34.37′', longitude: 88° 15′ 47.68′') 3. Village: Dole para, Ward:09, Union: Maharajpur, Sadar, Chapainawabganj (Latitude: 24° 37′ 9.93′', longitude: 88° 13′ 39.46′') 4. Village: Ramchandrapur hat, Ward:08, Union: Dohilpara, Sadar, Chapainawabganj (Latitude: 24° 36′ 27.83″, longitude: 88° 12′ 7.25″)
Data accessibility	Repository name: Mendeley Data [8] Data identification number: 10.17632/x4hgnjj5gv.2 Direct URL to data: https://data.mendeley.com/datasets/x4hgnjj5gv/2

## 1 Value of the Data

• 741 images of arsenic-affected people from Bangladesh and developed the first-ever image dataset of arsenic-affected people are captured manually with the camera of smartphones. Additionally, 741 images of non-arsenic affected people are also captured to cover both affected and non-affected people.
• After doing augmentation of the dataset, the size of the total dataset reaches 8892. In this dataset for both non-arsenic-affected people and arsenic-affected people, 4446 images have been collected.The dataset has a size of 3.60 GB and includes 1482 high-quality images . These images are captured with four different smartphones having resolutions to introduce variety in the dataset.
• Different methods for data validation are employed to convert the initial raw dataset into a refined version. These techniques include procedures such as eliminating noise, human-assisted labeling to establish ground truth, resizing images, as well as applying zoom and rotation transformations [1,7]. Factors such as image quality, severity of the illness, and demographic elements are being carefully considered.
• This dataset will be able to serve researchers in the field of dermatology to detect diseases caused by arsenic. By developing a freely available dataset and supporting developments in deep learning-based diagnostic tools, this dataset will be able to minimize this gap. By making a standardized dataset for arsenicosis detection available, this work aims to contribute to research and help those who are afflicted.

## 2 Data Description

This section describes the details of the images inside the dataset that hold the research information as well as the structures of the folders in the data repository.

### 2.1 Dataset details

The captured images in the dataset contain 8892 images, that is, 4446 images for both affected skin and healthy skin. To prepare each image for further investigation like- machine learning, deep learning, or image processing those are converted into a resolution of 244p × 244p. All the image files in the dataset are encoded in standard PNG format.

To differentiate the inter-class feature vectors during the prediction phase effectively, the types from different classes of the dataset certainly have distinguishing characteristics. It helps AI models to predict different types of arsenic in different body parts effectively [5]. In this part, the unique characteristics of numerous illnesses identified in the arsenic images of the dataset have been examined. In the following figures, two representative photos of each category, the left side shows the part of a body affected by arsenic and the right side shows the images of skin in good condition is shown.

**Fig. 1:**
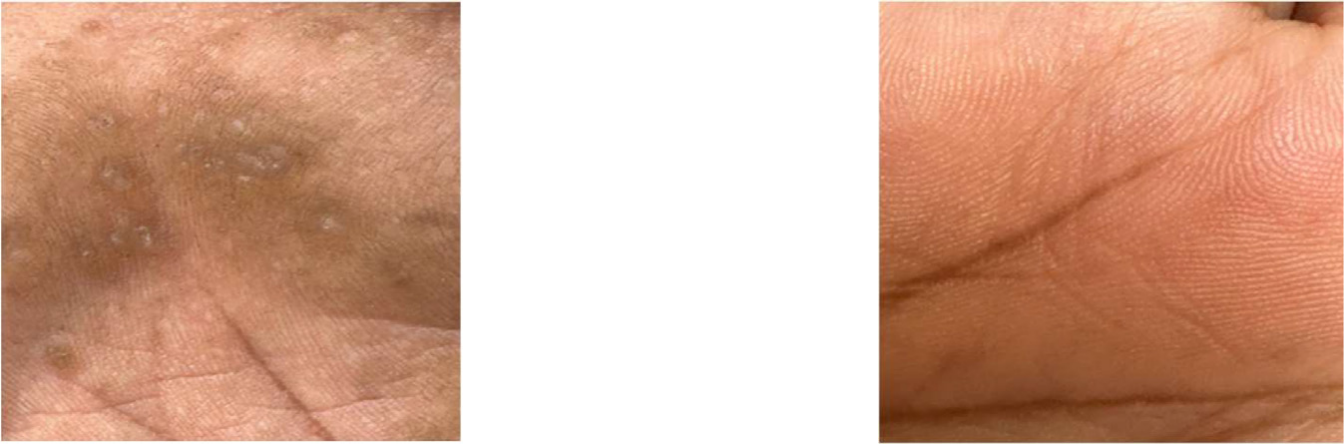
The palm of an affected person on the left and the palm of a healthy person on the right.

**Fig. 2:**
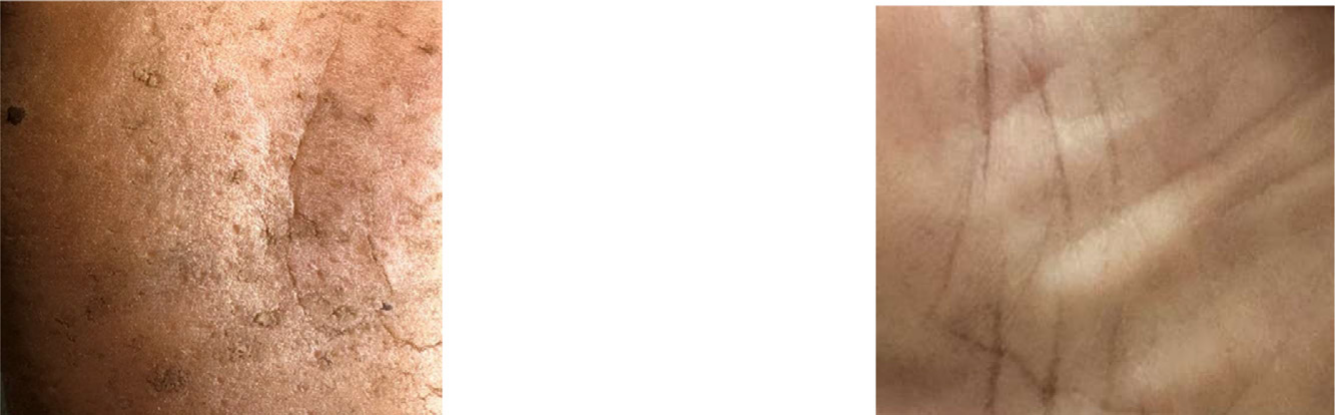
The sole of an affected person on the left and the sole of a healthy person on the right.

In cases of arsenicosis, noticeable symptoms of the illness can manifest on both the hands and feet. Some of these symptoms include conditions such as melanosis, hyperkeratosis, and hyperpigmentation. Hyperkeratosis is characterized by the development of rough and thicker areas on the skin, while hyperpigmentation refers to the abnormal darkening of the skin. Melanosis is indicated by the presence of darkened patches on the skin [11]. This visual representation can be observed in Figs. 1, and 2, where the distinct characteristics of these skin effects caused by arsenic poisoning are evident on both the hands and feet.

**Fig. 3:**
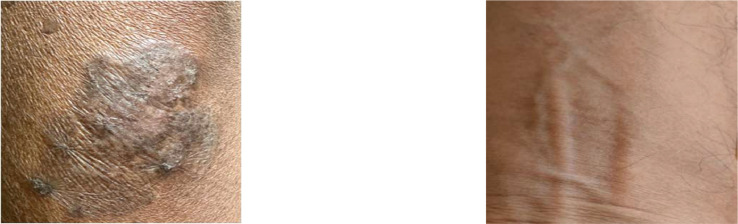
The arm of an affected person on the left and the arm of a healthy person on the right.

**Fig. 4:**
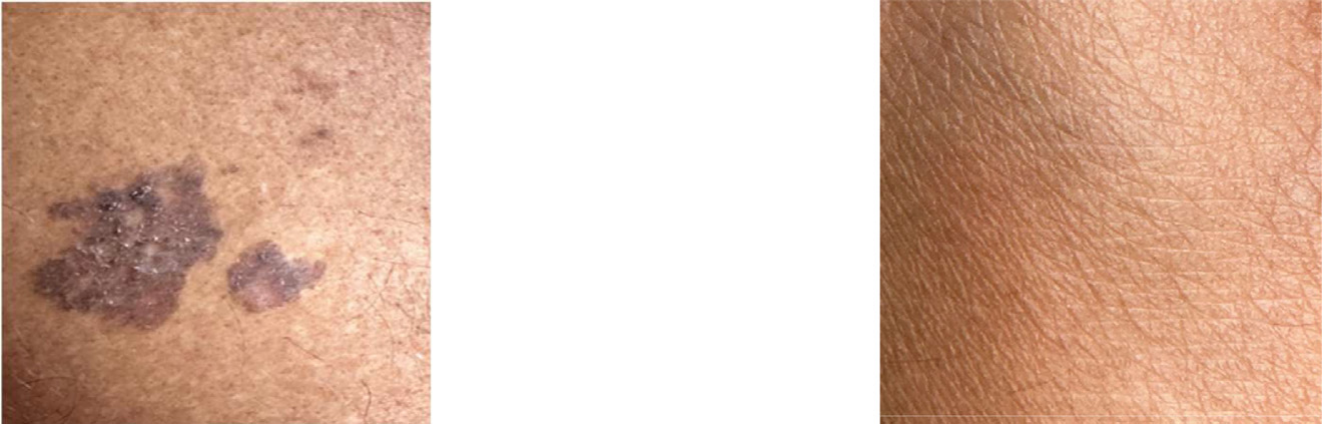
The skin of an affected person on the left and the skin of a healthy person on the right.

Individuals affected by arsenicosis may exhibit specific skin traits like melanosis, hyperkeratosis, and hyperpigmentation [4]. The intensity of arsenic exposure causes dark spots or marks on that particular affected person. They are represented by unusual darkened patches, rough and thicker skin areas, and the appearance of darker spots, as illustrated in Figs. 3 and 4. For a concise overview of the dataset, Table 1 is provided below, which provides summarized information about these skin conditions.

**Table 1:** ArsenicSkinImageBD dataset information at a glance.

Type of data	Image file (Dimension: 244p × 244p)
Data format	PNG
Number of images	The dataset contains 8892 images in total, of which 1482 are original skin images. The remaining images were created by applying augmentation techniques to the original images.
Diseases considered	Arsenicosis
Number of classes	Two (affected and healthy).
Distribution of instances	Both the affected and the healthy category has 4446 images.
How data are acquired	Human subjects were photographed using four smartphones with different camera specifications.
Data source locations	1. Village: Betbaria, Ward:08, Union: Balidanga, 2. Village: Balubagan, Ward:08, Union: Maharajpur, 3. Village: Dole para, Ward:09, Union: Maharajapur, 4. Village: Ramchandrapur hat, Ward:08, Union: Dohilpara, Sadar, Chapainawabganj.
Where applicable	Suitable to distinguish affected and healthy skin.

### 2.2 Dataset folder structure

**Fig. 5:**
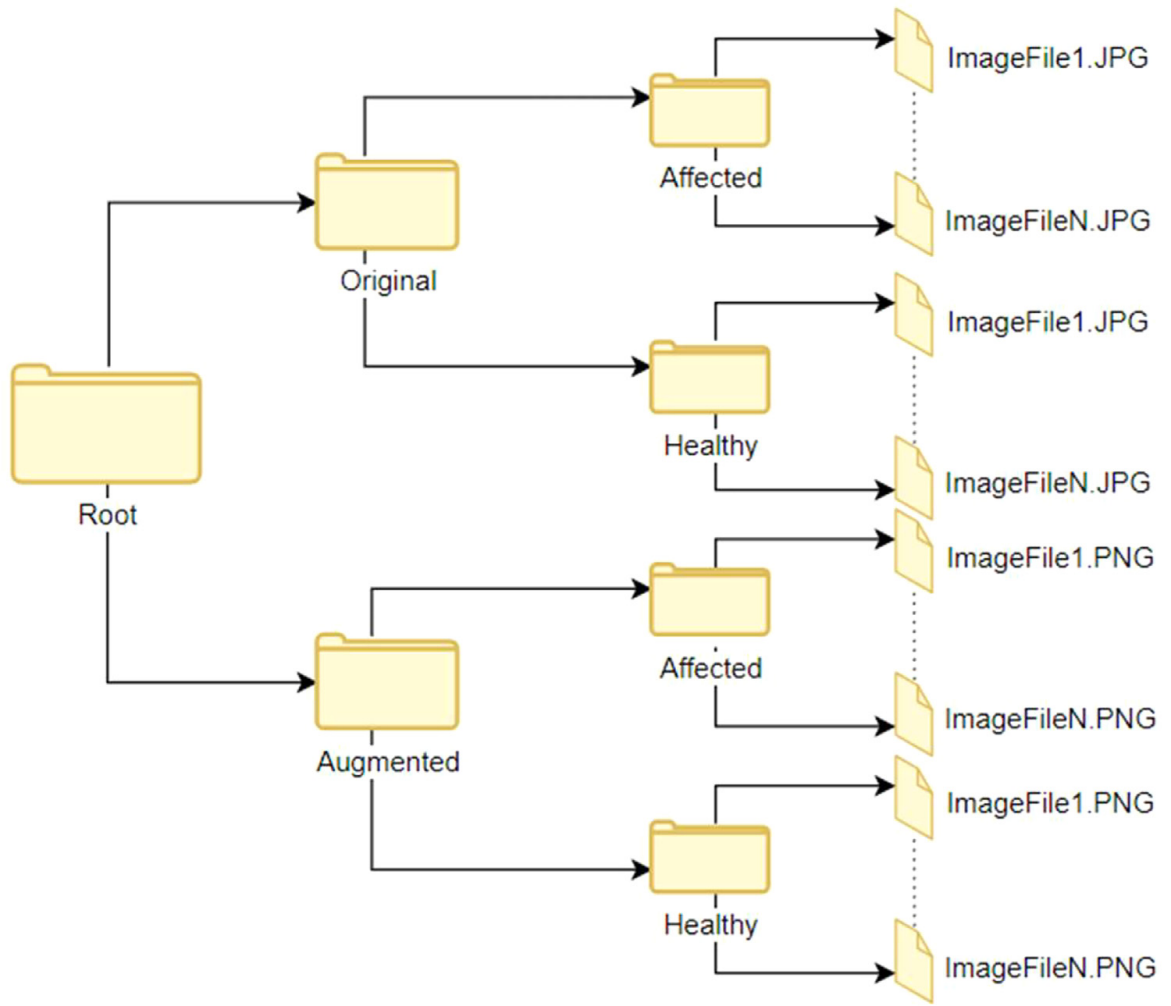
Overview of the directories of the dataset.

The root directory in the repository comprises two main folders, namely "Original" and "Augmented." Each of these folders contains two sub-folders with the following names: “Affected” and “Healthy”. Each of these sub-folders of the "Original" folder contains 741 high-quality images, while for “Augmented” folders the numbers are 4446. Fig. 5 illustrates the folder and sub-folder structure that categorizes the images.

## 3 Experimental Design, Materials and Methods

Lately, the rapid acceleration of technological advancement has led to the utilization of technologies for assisting humans in the effective diagnosis of various skin diseases in a relatively shorter time and at reduced costs. The application of technologies has been enabled by the advancements. Artificial Intelligence provides various technologies that are known for their capabilities to predict future events given relevant historical data and has gained prominence due to its accurate forecasting abilities. Successful applications of image processing algorithms in various sectors, including dermatology, have been facilitated using affordable computer hardware [5,7]. Machine learning techniques were employed on skin images to diagnose various diseases with high accuracy, achieving up to a 95 % success rate. The achievement of this accuracy can be attributed to the utilization of appropriate and robust datasets. The core of any artificial intelligence-based algorithm lies in the collection of a high-quality dataset, which is subsequently employed for model training and testing. Using this trained model, the algorithms help to find different patterns and insights into those patterns [[1], [9]].

Emphasizing the necessity for a diverse range of datasets to enable the prediction of diversified results, the significance of the dataset is of great importance. The purpose of this study is to establish a standardized and easily accessible dataset of skin images from Bangladesh. Bangladesh is struggling with a severe public health crisis, characterized by widespread arsenic poisoning, a concern that the World Health Organization (WHO) has labeled as the "largest mass poisoning of a population in history". To our knowledge, there is currently no publicly available dataset specifically encompassing skin images related to arsenic contamination in Bangladesh, inclusive of accurate labels denoting the presence of skin infections.

### 3.1 Steps of dataset collection and preparation

The dataset preparation process requires researchers to follow best practices rigorously and focus on data quality. Each image was carefully labeled by experienced human experts. Afterward, all the images were made sure to have the same size and shape, which is important for machine learning analysis. Finally, any unwanted background noise was removed from the images. The primary steps within the entire process of preparing the dataset include the following activities:

1. To acquire relevant information regarding the occurrence and attributes of skin diseases resulting from arsenic exposure.
2. To locate an ideal region where arsenic contamination is severe among the inhabitants.
3. To choose individuals who have been affected by arsenicosis for the image data collection.
4. To capture the images of both skin lesions affected by arsenicosis and normal skin lesions.
5. To implement validation techniques to ensure the accuracy and usability of the dataset images.

**Fig. 6:**
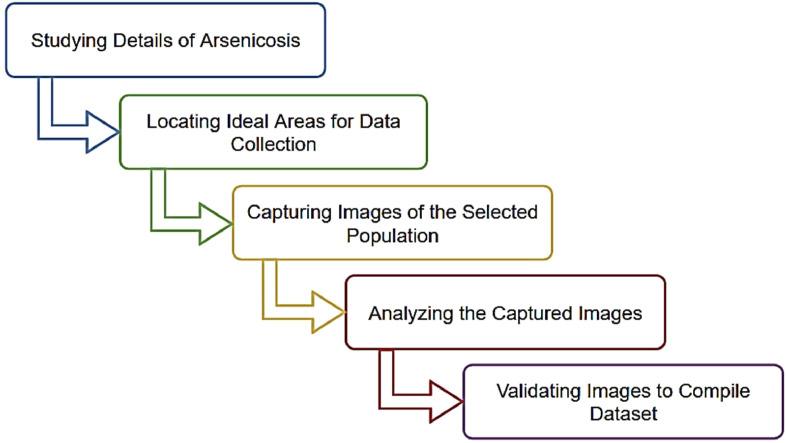
Methodology of ArsenicSkinImageBD dataset preparation.

Fig. 6 shows a flowchart of the methodology of the ArsenicSkinImageBD dataset preparation. The following sections describe each of the steps taken to gather and carefully annotate the arsenic skin disease dataset in detail.

### 3.2 Studying the details of arsenicosis

Arsenic, an element that belongs to Group 15 [Va] of the periodic table, is considered a significant environmental toxin with detrimental effects on human health [6]. Two distinct crystalline forms, grey and yellow, exist for arsenic; however, only the grey variant holds industrial significance. Notably, it has been assigned the highest priority on the list of the USA, as designated by the Agency for Toxic Substances and Disease Registry (ASTDR) until the year 2020 [2]. Recent estimations from reports indicate that a substantial number of individuals in various countries, including India, Bangladesh, and Pakistan, are exposed to arsenic concentrations in their water that surpass the recommended limit of 10 mg/L set by the World Health Organization. In this sub-continent, the approximate amount of contaminated people is respectively 28–60 million in Bangladesh, 47–60 million in Pakistan, and 70–80 million in India [12].

For individuals who are contaminated by arsenic, the most prevalent skin abnormalities include hyperkeratosis, hyperpigmentation, and skin cancer. These abnormalities can appear on any part of the body, often manifesting as irregular areas of increased pigmentation. Prolonged arsenic exposure can lead to patchy hyperpigmentation, frequently observed in regions such as the axillae, eyes, crotch, neck, nipples, and temples. The unique presentation, characterized by dark brown patches accompanied by scattered pale spots, is sometimes likened to the appearance of "raindrops on a sandy road." In severe cases, the pigmentation can extend extensively across the hands, legs, belly, back, and chest [4].

### 3.3 Locating the ideal region for the data collection

A careful process was followed to select the specific area in Chapainawabganj, Bangladesh, where a large population was affected by a high prevalence of arsenic poisoning. The largest village in the region with a significant number of people showing symptoms of arsenic poisoning across different segments was chosen [7]. This method aimed to concentrate on the current cases of people suffering from arsenic poisoning. The selection process also accounted for the various locations within the region that were affected to ensure a representative sample. Hence, the selected villages from Chapainawabganj are Betbaria, Balubagan, Dole para, and Ramchandrapur hat.

The Daily Star news also confirmed that The Department of Public Health had done its research to find areas with arsenic-contaminated water [3]. Furthermore, medical professionals verified the existence of people with arsenic poisoning in the area. To reduce potential biases in the collected data, a diverse range of locations within Chapainawabganj was carefully selected. By doing this, the research intends to capture a comprehensive and diverse representation of the impact of arsenic poisoning [11].

### 3.4 Selecting the arsenic affected patients

Serious health issues have been brought about by the arsenic problem in Bangladesh, affecting numerous individuals and communities. Therefore, a respectful and compassionate approach is necessary when discussing this matter. Painful memories might be triggered for some individuals by the act of capturing images. Sharing their photos with strangers was something most villagers were sensitive about and disinclined to do. Concerns were also raised about the potential misuse of the photos for unlawful purposes. The participants were informed about the purpose of the photography session and the intended use of the photos, which contributed to their comfort and understanding of the process. Consent was also sought from them before photographing, a step that was vital to ensure their awareness of the photo usage and their agreement to be photographed. Collaboration was established with the Department of Public Health to oversee the photography session. This involvement meant that relevant authorities or the government were engaged, lending credibility to the process and ensuring adherence to appropriate regulations and standards.

### 3.5 Capturing images of the selected subjects

In April 2023, a photo capture session was conducted to get images from the people of the selected area. The purpose of this collection was to build a dataset for training any AI models. To ensure accuracy and ethical considerations, a structured approach was followed. Individuals who were known to be affected by arsenic contamination in Chapainawabganj were specifically targeted [10]. Images were captured using four smartphones with different camera resolutions. Table 2 shows the details of the devices using which the images were captured. All the devices were used to capture the images of healthy skins but for the affected skin, only iPhone 14 Pro max was used.

**Table 2:** Details of the devices for data collection.

Device	Camera	Resolution
Huawei Y9	13 megapixels	1080p × 2340p
POCO X3	64 megapixels	1080p × 2400p
Oppo Reno 6	64 megapixels	1080p × 2340p
iPhone 14 Pro-max	48 megapixels	1290p × 2796p

The session took place on a sunny day during the summer season, ensuring optimal lighting conditions without any interference from clouds or fog. Each participant's affected area was photographed separately to ensure clarity and accuracy. After the completion of the photography session, 1482 images were collected. These images displayed two distinct categories: healthy skin and skin affected by arsenic contamination. The detailed identification of the subject is not disclosed due to ethical reasons. Instead, we have provided an identification number to specify the individuals. Table 3 holds the information by which one can map the individual arsenic-affected subjects with their captured images. The first column represents the subject ID, the second column denotes the specific village of the subject, and the third column contains labels for all images collected from that particular individual. The complete information of this table can be found in the readme file of the data repository [8].

**Table 3:** Image information of arsenic infected subjects.

S. ID	Village name	Labels of images
10,001	Village: Betbaria, Ward:08, Union: Balidanga, Sadar, Chapainawabganj	IMG_1169.JPG IMG_1170.JPG IMG_1171.JPG …….. IMG_1185.JPG IMG_1186.JPG
10,002	Village: Betbaria, Ward:08, Union: Balidanga, Sadar, Chapainawabganj	IMG_1342.JPG IMG_1343.JPG IMG_1344.JPG ……. IMG_1369.JPG IMG_1370.JPG
100,037	Village: Ramchandra, Ward:08, Union: Dohilpara, Sadar, Chapainawabganj	IMG_1314.JPG IMG_1315.JPG IMG_1316.JPG ……. IMG_1339.JPG IMG_1340.JPG

### 3.6 Validating the images of the dataset

**Fig. 7:**
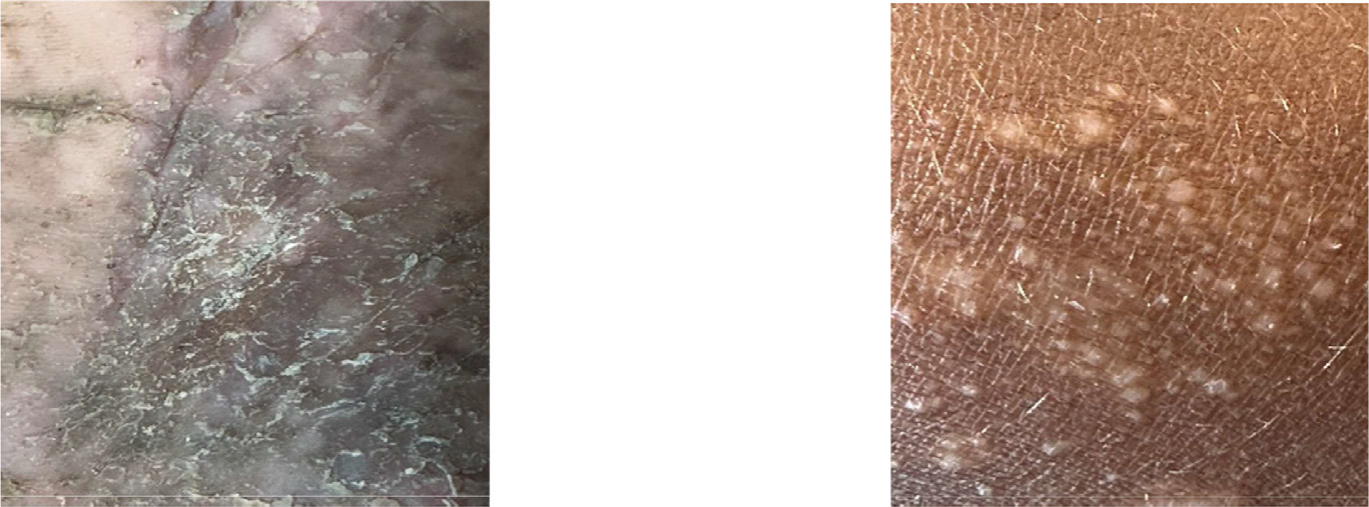
The images of the skin of non-arsenic-affected subjects having a resemblance.

Several steps were performed to make the dataset ready for machine learning analysis. The size of each image was standardized to 244p × 244p pixels. This step made the image dimensions consistent, which helped train machine learning models. The images were saved in PNG format to preserve visual quality and enable further processing. The images were checked carefully, and any blurry, out-of-focus, and duplicate images were removed from the dataset. The final dataset had about 8892 images, with 4446 images for the healthy skin class and for the skin affected by arsenic. With a view to adding a layer of complexity to the dataset, some images of skin that resembled arsenic-affected skin but were not actually contaminated are also included. Fig. 7 shows a sample of that kind of image and the detailed list can be found can be found in the readme file of the data repository [8].

This inclusion aimed to train the AI models to differentiate between true arsenic contamination and similar-looking conditions that are not caused by arsenic. During the preparation of the dataset, the original images from both categories were confirmed by an expert familiar with identifying arsenic-affected skin to eliminate any wrong information. The labels accurately indicated whether each image depicted healthy skin or skin with similar appearances but no actual contamination, and arsenic-affected skin.

## Limitations

• Some people may be reluctant to provide their images for research because they are concerned about privacy, how their images might be used, or for personal reasons. If a large number of people choose not to participate, this could lead to a smaller dataset. Additionally, if those who decline to participate differ from those who agree in terms of demographics, socioeconomic status, or health factors, this could introduce bias into the dataset. The resulting dataset may not accurately represent the entire population being studied, which could impact the validity of the findings.
• Arsenic contamination can have serious health implications, and those affected may have experienced physical and emotional trauma. This sensitivity could lead to self-selection bias, where people with more severe or noticeable symptoms of contamination may be more or less likely to participate in the photo capture session. This bias could impact the dataset's representation of the range of arsenic contamination effects, potentially skewing the analysis and findings.
• The use of different smartphones with varying camera resolutions and the influence of environmental conditions like lighting can introduce variability in the images captured. This variability can lead to differences in image quality, clarity, and color accuracy. These inconsistencies can introduce noise into the dataset, making it harder to discern meaningful patterns.
• The findings of this study may not be applicable to other regions or communities with different arsenic contamination levels or socio-economic contexts. Arsenic contamination can vary in prevalence and impact across different regions. Socio-economic factors, cultural practices, and local governance can also influence the contamination's effects and responses. Therefore, while the study's findings may be valuable for the specific context studied, they may not readily translate to other settings.

## Ethics Statement

The procedure for gathering data involved the participation of human subjects and adhering to the ethical guidelines outlined in the Declaration of Helsinki by the World Medical Association. All individuals taking part were educated about the research's purpose and granted their consent. Additionally, the significance of the research was explained to them before collecting any data. Special care was taken to ensure the privacy of elders, minors, and female participants.

## CRediT authorship contribution statement

**Ismot Ara Emu:** Methodology, Investigation, Data curation, Writing – original draft. **Nishat Tasnim Niloy:** Investigation, Data curation, Formal analysis, Writing – review & editing. **Bhuyan Md Anowarul Karim:** Writing – original draft, Methodology, Data curation. **Anindya Chowdhury:** Writing – original draft, Investigation, Data curation. **Fatema Tuj Johora:** Writing – original draft, Data curation. **Mahamudul Hasan:** Visualization, Validation. **Tanni Mittra:** Methodology, Data curation. **Mohammad Rifat Ahmmad Rashid:** Methodology, Investigation. **Taskeed Jabid:** Visualization, Data curation. **Maheen Islam:** Project administration, Investigation. **Md. Sawkat Ali:** Conceptualization, Methodology, Supervision, Project administration, Validation.

## Data Availability

ArsenicSkinImageBD (Original data) (Mendeley Data)

## Appendix Supplementary Materials

Image, 1

Image, application 2
