# Supplementary figures and images for: ArsenicSkinImageBD: A comprehensive image dataset to classify affected and healthy skin of arsenic-affected people
Source: Data Brief. 2023 Dec 28;52:110016. doi: 10.1016/j.dib.2023.110016 (PMC10827410; doi:10.1016/j.dib.2023.110016)

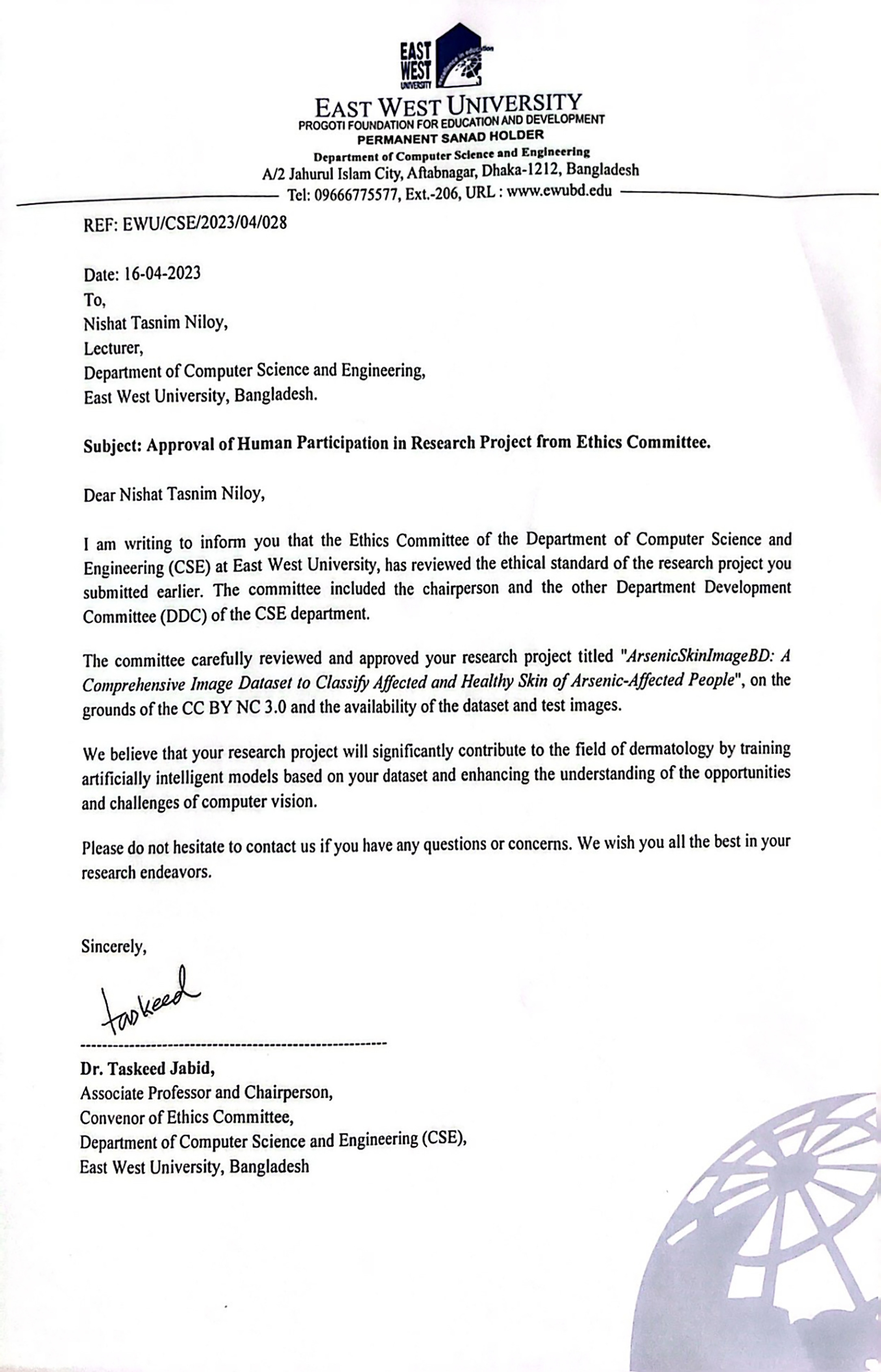

Supplement: Supplementary file 1 [file mmc1.jpg]
