# Supplementary material for: ArsenicSkinImageBD: A comprehensive image dataset to classify affected and healthy skin of arsenic-affected people
Source: Data Brief. 2023 Dec 28;52:110016. doi: 10.1016/j.dib.2023.110016 (PMC10827410; doi:10.1016/j.dib.2023.110016)
Supplement: Supplementary file 2 [file mmc2.docx]

# Summary of the Dataset

In the vast realm of data-driven research, the effective organization and annotation of image datasets play a pivotal role in extracting meaningful insights. Therefore, we created an annotation file to map the images of the dataset, with the subject from which we have collected the dataset. This document focuses on the structure of the folders, identification of the subject's unique identifier, referred to as the Subject ID, the recording of the geographical origin of the subject, specifically the village from which it was obtained, and the list of skins that are not arsenic affected but have resemblances.

## **Structure of the folders of the Dataset**

From the root folder, we can find two folders- Original and Augmented. Both of these folders share the same structures. There are two folders- Affected and Healthy. Inside these folders, the images can be found. As for the “Original” directory, each of the Affected and Healthy contains 741 images, and in the “Augmented” director, the numbers are 4446. Figure 1 depicts the total illustration of the structure.


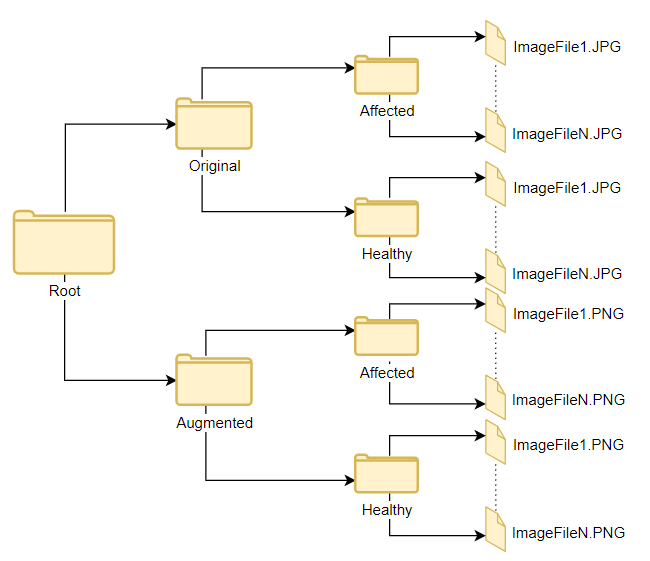


Figure 1: Structure of the Dataset Directory

## **Mapping the Subject IDs, Their Location, and Labels of the Images from the Dataset**

The detailed identification of the subject is not disclosed due to ethical reasons. Instead, we have provided an identification number to specify the individuals. Table 1 holds the information on arsenic-infected patients’ images. The first column represents the subject ID, the second column denotes the specific village of the subject, and the third column contains labels for all images collected from that particular individual. We collected 741 images from 37 affected individuals and another 741 from 76 healthy individuals.

Table 1: Image Information of Arsenic Infected Subjects

| S. ID | Village Name | Label |
| --- | --- | --- |
| 10001 | Village: Betbaria, Ward:08, Union: Balidanga, Sadar, Chapainawabganj | IMG_1169.JPG  IMG_1170.JPG  IMG_1171.JPG  IMG_1172.JPG  IMG_1173.JPG  IMG_1174.JPG  IMG_1175.JPG  IMG_1176.JPG  IMG_1177.JPG  IMG_1178.JPG  IMG_1179.JPG  IMG_1180.JPG  IMG_1181.JPG  IMG_1182.JPG  IMG_1183.JPG  IMG_1184.JPG  IMG_1185.JPG  IMG_1186.JPG |
| 10002 | Village: Betbaria, Ward:08, Union: Balidanga, Sadar, Chapainawabganj | IMG_1342.JPG  IMG_1343.JPG  IMG_1344.JPG  IMG_1341.JPG  IMG_1345.JPG  IMG_1346.JPG  IMG_1347.JPG  IMG_1348.JPG  IMG_1349.JPG  IMG_1350.JPG  IMG_1351.JPG  IMG_1352.JPG  IMG_1353.JPG  IMG_1354.JPG  IMG_1355.JPG  IMG_1356.JPG  IMG_1357.JPG  IMG_1358.JPG  IMG_1359.JPG  IMG_1360.JPG  IMG_1362.JPG  IMG_1363.JPG  IMG_1364.JPG  IMG_1365.JPG  IMG_1366.JPG  IMG_1367.JPG  IMG_1368.JPG  IMG_1369.JPG  IMG_1370.JPG |
| 10003 | Village: Betbaria, Ward:08, Union: Balidanga, Sadar, Chapainawabganj | IMG_1371.JPG  IMG_1372.JPG  IMG_1373.JPG  IMG_1374.JPG  IMG_1375.JPG  IMG_1376.JPG  IMG_1377.JPG  IMG_1378.JPG  IMG_1379.JPG  IMG_1380.JPG  IMG_1381.JPG  IMG_1382.JPG  IMG_1383.JPG  IMG_1384.JPG  IMG_1385.JPG  IMG_1386.JPG  IMG_1387.JPG  IMG_1388.JPG  IMG_1389.JPG  IMG_1390.JPG  IMG_1391.JPG  IMG_1392.JPG  IMG_1393.JPG  IMG_1394.JPG  IMG_1395.JPG  IMG_1396.JPG  IMG_1397.JPG  IMG_1398.JPG  IMG_1399.JPG  IMG_1400.JPG  IMG_1401.JPG  IMG_1402.JPG  IMG_1403.JPG  IMG_1404.JPG  IMG_1405.JPG  IMG_1406.JPG  IMG_1407.JPG  IMG_1408.JPG  IMG_1409.JPG  IMG_1410.JPG  IMG_1411.JPG |
| 10004 | C Village: Betbaria, Ward:08, Union: Balidanga, Sadar, Chapainawabganj | IMG_1412.JPG  IMG_1413.JPG  IMG_1414.JPG  IMG_1415.JPG  IMG_1416.JPG  IMG_1417.JPG  IMG_1418.JPG  IMG_1419.JPG  IMG_1420.JPG  IMG_1421.JPG  IMG_1422.JPG  IMG_1423.JPG |
| 10005 | Village: Betbaria, Ward:08, Union: Balidanga, Sadar, Chapainawabganj | IMG_1424.JPG  IMG_1425.JPG  IMG_1426.JPG  IMG_1427.JPG  IMG_1428.JPG  IMG_1429.JPG  IMG_1430.JPG  IMG_1431.JPG  IMG_1432.JPG  IMG_1433.JPG  IMG_1434.JPG  IMG_1435.JPG  IMG_1436.JPG  IMG_1437.JPG  IMG_1438.JPG  IMG_1439.JPG  IMG_1440.JPG  IMG_1441.JPG  IMG_1442.JPG |
| 10006 | Village: Betbaria, Ward:08, Union: Balidanga, Sadar, Chapainawabganj | IMG_1443.JPG  IMG_1444.JPG  IMG_1445.JPG  IMG_1446.JPG  IMG_1447.JPG |
| 10007 | Village: Betbaria, Ward:08, Union: Balidanga, Sadar, Chapainawabganj | IMG_1448.JPG  IMG_1449.JPG  IMG_1450.JPG  IMG_1451.JPG  IMG_1452.JPG  IMG_1453.JPG  IMG_1454.JPG  IMG_1455.JPG  IMG_1456.JPG  IMG_1457.JPG  IMG_1458.JPG  IMG_1459.JPG  IMG_1460.JPG  IMG_1461.JPG  IMG_1462.JPG  IMG_1463.JPG  IMG_1464.JPG  IMG_1465.JPG  IMG_1466.JPG  IMG_1467.JPG  IMG_1468.JPG  IMG_1469.JPG  IMG_1470.JPG  IMG_1471.JPG  IMG_1472.JPG |
| 10008 | Village: Betbaria, Ward:08, Union: Balidanga, Sadar, Chapainawabganj | IMG_1474.JPG  IMG_1475.JPG  IMG_1476.JPG  IMG_1477.JPG  IMG_1478.JPG  IMG_1479.JPG  IMG_1480.JPG  IMG_1481.JPG  IMG_1482.JPG  IMG_1483.JPG  IMG_1484.JPG  IMG_1485.JPG  IMG_1486.JPG  IMG_1487.JPG  IMG_1488.JPG  IMG_1489.JPG  IMG_1490.JPG  IMG_1491.JPG |
| 10009 | Village: Betbaria, Ward:08, Union: Balidanga, Sadar, Chapainawabganj | IMG_1492.JPG  IMG_1493.JPG  IMG_1494.JPG  IMG_1495.JPG  IMG_1496.JPG  IMG_1497.JPG  IMG_1498.JPG  IMG_1499.JPG  IMG_1500.JPG  IMG_1501.JPG  IMG_1502.JPG  IMG_1503.JPG  IMG_1504.JPG  IMG_1505.JPG  IMG_1506.JPG  IMG_1507.JPG |
| 10010 | Village: Betbaria, Ward:08, Union: Balidanga, Sadar, Chapainawabganj | IMG_1508.JPG  IMG_1509.JPG  IMG_1510.JPG  IMG_1511.JPG  IMG_1512.JPG  IMG_1513.JPG  IMG_1514.JPG  IMG_1515.JPG  IMG_1516.JPG  IMG_1517.JPG  IMG_1518.JPG  IMG_1519.JPG  IMG_1520.JPG  IMG_1521.JPG  IMG_1522.JPG  IMG_1523.JPG  IMG_1524.JPG  IMG_1525.JPG  IMG_1526.JPG  IMG_1527.JPG  IMG_1528.JPG  IMG_1529.JPG  IMG_1530.JPG  IMG_1531.JPG  IMG_1532.JPG  IMG_1533.JPG |
| 10011 | Village: Betbaria, Ward:08, Union: Balidanga, Sadar, Chapainawabganj | IMG_1534.JPG  IMG_1535.JPG  IMG_1536.JPG  IMG_1537.JPG  IMG_1538.JPG  IMG_1539.JPG  IMG_1540.JPG  IMG_1541.JPG  IMG_1542.JPG  IMG_1543.JPG  IMG_1544.JPG  IMG_1545.JPG  IMG_1546.JPG  IMG_1547.JPG  IMG_1548.JPG  IMG_1549.JPG  IMG_1550.JPG  IMG_1551.JPG  IMG_1552.JPG  IMG_1553.JPG  IMG_1554.JPG  IMG_1555.JPG  IMG_1556.JPG  IMG_1557.JPG  IMG_1558.JPG  IMG_1559.JPG  IMG_1560.JPG  IMG_1561.JPG  IMG_1562.JPG |
| 10012 | Village: Balubagan, Ward:08, Union: Maharajpur, Sadar, Chapainawabganj | IMG_1563.JPG  IMG_1564.JPG  IMG_1565.JPG  IMG_1566.JPG  IMG_1567.JPG  IMG_1568.JPG  IMG_1569.JPG  IMG_1570.JPG  IMG_1571.JPG  IMG_1572.JPG  IMG_1573.JPG  IMG_1574.JPG  IMG_1575.JPG  IMG_1576.JPG  IMG_1577.JPG  IMG_1578.JPG  IMG_1579.JPG  IMG_1580.JPG  IMG_1581.JPG  IMG_1582.JPG  IMG_1583.JPG  IMG_1584.JPG  IMG_1585.JPG  IMG_1586.JPG  IMG_1587.JPG  IMG_1588.JPG  IMG_1589.JPG  IMG_1590.JPG  IMG_1591.JPG  IMG_1592.JPG  IMG_1593.JPG  IMG_1594.JPG  IMG_1595.JPG  IMG_1596.JPG  IMG_1597.JPG  IMG_1598.JPG  IMG_1599.JPG  IMG_1600.JPG |
| 10013 | Village: Balubagan, Ward:08, Union: Maharajpur, Sadar, Chapainawabganj | IMG_1187.JPG  IMG_1188.JPG  IMG_1189.JPG  IMG_1190.JPG  IMG_1191.JPG  IMG_1192.JPG  IMG_1193.JPG  IMG_1194.JPG  IMG_1361.JPG |
| 10014 | Village: Balubagan, Ward:08, Union: Maharajpur, Sadar, Chapainawabganj | IMG_1601.JPG  IMG_1602.JPG  IMG_1603.JPG  IMG_1604.JPG  IMG_1605.JPG  IMG_1606.JPG  IMG_1607.JPG  IMG_1608.JPG  IMG_1609.JPG  IMG_1610.JPG  IMG_1611.JPG  IMG_1612.JPG  IMG_1613.JPG  IMG_1614.JPG  IMG_1615.JPG  IMG_1616.JPG  IMG_1617.JPG |
| 10015 | Village: Balubagan, Ward:08, Union: Maharajpur, Sadar, Chapainawabganj | IMG_1618.JPG  IMG_1619.JPG  IMG_1620.JPG  IMG_1621.JPG  IMG_1622.JPG  IMG_1623.JPG  IMG_1624.JPG  IMG_1625.JPG  IMG_1626.JPG  IMG_1627.JPG  IMG_1628.JPG  IMG_1629.JPG  IMG_1630.JPG  IMG_1631.JPG  IMG_1632.JPG  IMG_1633.JPG  IMG_1634.JPG |
| 10016 | Village: Balubagan, Ward:08, Union: Maharajpur, Sadar, Chapainawabganj | IMG_1635.JPG  IMG_1636.JPG  IMG_1637.JPG  IMG_1638.JPG  IMG_1639.JPG  IMG_1640.JPG  IMG_1641.JPG  IMG_1642.JPG  IMG_1643.JPG  IMG_1644.JPG  IMG_1645.JPG  IMG_1646.JPG  IMG_1647.JPG  IMG_1648.JPG  IMG_1649.JPG |
| 10017 | Village: Balubagan, Ward:08, Union: Maharajpur, Sadar, Chapainawabganj | IMG_1650.JPG  IMG_1651.JPG  IMG_1652.JPG  IMG_1653.JPG  IMG_1654.JPG  IMG_1655.JPG  IMG_1656.JPG  IMG_1657.JPG  IMG_1658.JPG  IMG_1659.JPG  IMG_1660.JPG  IMG_1661.JPG  IMG_1662.JPG  IMG_1663.JPG  IMG_1664.JPG  IMG_1665.JPG  IMG_1666.JPG  IMG_1667.JPG  IMG_1668.JPG  IMG_1669.JPG  IMG_1670.JPG  IMG_1671.JPG  IMG_1672.JPG  IMG_1673.JPG  IMG_1674.JPG  IMG_1675.JPG |
| 10018 | Village: Balubagan, Ward:08, Union: Maharajpur, Sadar, Chapainawabganj | IMG_1676.JPG  IMG_1677.JPG  IMG_1678.JPG  IMG_1679.JPG  IMG_1680.JPG  IMG_1681.JPG  IMG_1682.JPG  IMG_1683.JPG  IMG_1684.JPG  IMG_1685.JPG  IMG_1686.JPG  IMG_1687.JPG  IMG_1688.JPG  IMG_1689.JPG  IMG_1690.JPG  IMG_1691.JPG  IMG_1692.JPG  IMG_1693.JPG  IMG_1694.JPG  IMG_1695.JPG  IMG_1696.JPG  IMG_1697.JPG  IMG_1698.JPG  IMG_1699.JPG  IMG_1700.JPG  IMG_1701.JPG |
| 10019 | Village: Balubagan, Ward:08, Union: Maharajpur, Sadar, Chapainawabganj | IMG_1702.JPG  IMG_1703.JPG  IMG_1704.JPG  IMG_1705.JPG  IMG_1706.JPG  IMG_1707.JPG  IMG_1708.JPG  IMG_1709.JPG  IMG_1710.JPG  IMG_1711.JPG  IMG_1712.JPG  IMG_1713.JPG  IMG_1714.JPG  IMG_1715.JPG  IMG_1716.JPG  IMG_1717.JPG  IMG_1718.JPG  IMG_1719.JPG  IMG_1720.JPG  IMG_1721.JPG  IMG_1722.JPG  IMG_1723.JPG  IMG_1724.JPG  IMG_1725.JPG  IMG_1726.JPG  IMG_1727.JPG  IMG_1728.JPG  IMG_1729.JPG  IMG_1730.JPG  IMG_1731.JPG  IMG_1732.JPG  IMG_1733.JPG  IMG_1734.JPG  IMG_1735.JPG  IMG_1736.JPG  IMG_1737.JPG  IMG_1738.JPG |
| 10020 | Village: Balubagan, Ward:08, Union: Maharajpur, Sadar, Chapainawabganj | IMG_1739.JPG  IMG_1740.JPG  IMG_1741.JPG  IMG_1742.JPG  IMG_1743.JPG  IMG_1744.JPG  IMG_1745.JPG  IMG_1746.JPG  IMG_1747.JPG  IMG_1748.JPG  IMG_1749.JPG  IMG_1750.JPG  IMG_1751.JPG  IMG_1752.JPG  IMG_1753.JPG  IMG_1754.JPG  IMG_1755.JPG  IMG_1756.JPG  IMG_1757.JPG  IMG_1758.JPG  IMG_1759.JPG  IMG_1760.JPG |
| 10021 | Village: Dole para, Ward:09, Union: Maharajpur, Sadar, Chapainawabganj | IMG_1761.JPG  IMG_1762.JPG  IMG_1763.JPG  IMG_1764.JPG  IMG_1765.JPG  IMG_1766.JPG  IMG_1767.JPG  IMG_1768.JPG  IMG_1769.JPG  IMG_1770.JPG  IMG_1771.JPG |
| 10022 | Village: Dole para, Ward:09, Union: Maharajpur, Sadar, Chapainawabganj | IMG_1772.JPG  IMG_1773.JPG  IMG_1774.JPG  IMG_1775.JPG  IMG_1776.JPG  IMG_1777.JPG  IMG_1778.JPG  IMG_1779.JPG  IMG_1780.JPG  IMG_1781.JPG  IMG_1782.JPG  IMG_1796.JPG  IMG_1804.JPG  IMG_1805.JPG  IMG_1806.JPG |
| 10023 | Village: Dole para, Ward:09, Union: Maharajpur, Sadar, Chapainawabganj | IMG_1783.JPG  IMG_1784.JPG  IMG_1785.JPG  IMG_1786.JPG  IMG_1787.JPG  IMG_1788.JPG  IMG_1789.JPG  IMG_1790.JPG  IMG_1791.JPG  IMG_1792.JPG  IMG_1793.JPG  IMG_1794.JPG  IMG_1795.JPG  IMG_1797.JPG |
| 10024 | Village: Dole para, Ward:09, Union: Maharajpur, Sadar, Chapainawabganj | IMG_1798.JPG  IMG_1799.JPG  IMG_1800.JPG  IMG_1801.JPG  IMG_1802.JPG  IMG_1803.JPG  IMG_1807.JPG  IMG_1808.JPG  IMG_1809.JPG  IMG_1810.JPG  IMG_1811.JPG  IMG_1812.JPG  IMG_1813.JPG |
| 10025 | Village: Dole para, Ward:09, Union: Maharajpur, Sadar, Chapainawabganj | IMG_1814.JPG  IMG_1815.JPG  IMG_1816.JPG  IMG_1817.JPG  IMG_1818.JPG  IMG_1819.JPG  IMG_1820.JPG  IMG_1821.JPG  IMG_1822.JPG  IMG_1823.JPG  IMG_1824.JPG  IMG_1825.JPG  IMG_1826.JPG  IMG_1827.JPG  IMG_1828.JPG  IMG_1829.JPG |
| 10026 | Village: Dole para, Ward:09, Union: Maharajpur, Sadar, Chapainawabganj | IMG_1830.JPG  IMG_1831.JPG  IMG_1832.JPG  IMG_1833.JPG  IMG_1834.JPG  IMG_1835.JPG  IMG_1836.JPG  IMG_1837.JPG  IMG_1838.JPG  IMG_1839.JPG  IMG_1840.JPG  IMG_1841.JPG |
| 10027 | Village: Dole para, Ward:09, Union: Maharajpur, Sadar, Chapainawabganj | IMG_1842.JPG  IMG_1843.JPG  IMG_1844.JPG  IMG_1845.JPG  IMG_1846.JPG  IMG_1847.JPG  IMG_1848.JPG  IMG_1849.JPG  IMG_1850.JPG  IMG_1851.JPG  IMG_1852.JPG  IMG_1853.JPG  IMG_1854.JPG  IMG_1855.JPG  IMG_1856.JPG  IMG_1857.JPG  IMG_1858.JPG  IMG_1859.JPG  IMG_1860.JPG  IMG_1861.JPG  IMG_1862.JPG  IMG_1863.JPG  IMG_1864.JPG  IMG_1865.JPG  IMG_1866.JPG  IMG_1867.JPG  IMG_1868.JPG  IMG_1869.JPG |
| 10028 | Village: Dole para, Ward:09, Union: Maharajpur, Sadar, Chapainawabganj | IMG_1870.JPG  IMG_1871.JPG  IMG_1872.JPG  IMG_1873.JPG  IMG_1874.JPG  IMG_1875.JPG  IMG_1876.JPG  IMG_1877.JPG  IMG_1878.JPG  IMG_1879.JPG  IMG_1880.JPG  IMG_1881.JPG  IMG_1882.JPG  IMG_1883.JPG  IMG_1884.JPG  IMG_1885.JPG  IMG_1886.JPG  IMG_1887.JPG  IMG_1888.JPG  IMG_1889.JPG  IMG_1890.JPG  IMG_1891.JPG |
| 10029 | Village: Dole para, Ward:09, Union: Maharajpur, Sadar, Chapainawabganj | IMG_1892.JPG  IMG_1893.JPG  IMG_1894.JPG  IMG_1895.JPG  IMG_1896.JPG  IMG_1897.JPG  IMG_1898.JPG  IMG_1899.JPG  IMG_1900.JPG  IMG_1901.JPG  IMG_1902.JPG  IMG_1903.JPG  IMG_1904.JPG  IMG_1905.JPG  IMG_1906.JPG  IMG_1907.JPG  IMG_1908.JPG  IMG_1909.JPG  IMG_1910.JPG  IMG_1911.JPG  IMG_1912.JPG  IMG_1913.JPG |
| 10030 | Village: Dole para, Ward:09, Union: Maharajpur, Sadar, Chapainawabganj | IMG_1195.JPG  IMG_1196.JPG  IMG_1197.JPG  IMG_1198.JPG  IMG_1199.JPG  IMG_1200.JPG  IMG_1201.JPG  IMG_1202.JPG  IMG_1203.JPG |
| 10031 | Village: Dole para, Ward:09, Union: Maharajpur, Sadar, Chapainawabganj | IMG_1204.JPG  IMG_1205.JPG  IMG_1206.JPG  IMG_1207.JPG  IMG_1208.JPG  IMG_1209.JPG  IMG_1210.JPG  IMG_1211.JPG  IMG_1212.JPG  IMG_1213.JPG  IMG_1215.JPG  IMG_1216.JPG  IMG_1217.JPG  IMG_1218.JPG  IMG_1219.JPG  IMG_1220.JPG  IMG_1221.JPG  IMG_1222.JPG  IMG_1223.JPG  IMG_1224.JPG  IMG_1225.JPG |
| 10032 | Village: Dole para, Ward:09, Union: Maharajpur, Sadar, Chapainawabganj | IMG_1226.JPG  IMG_1227.JPG  IMG_1228.JPG  IMG_1229.JPG  IMG_1230.JPG  IMG_1231.JPG  IMG_1232.JPG  IMG_1233.JPG  IMG_1234.JPG  IMG_1235.JPG  IMG_1236.JPG  IMG_1237.JPG  IMG_1238.JPG  IMG_1239.JPG  IMG_1240.JPG |
| 10033 | Village: Ramchandra, Ward:08, Union: Dohilpara, Sadar, Chapainawabganj | IMG_1241.JPG  IMG_1242.JPG  IMG_1243.JPG  IMG_1244.JPG  IMG_1245.JPG  IMG_1246.JPG  IMG_1247.JPG  IMG_1248.JPG  IMG_1249.JPG  IMG_1250.JPG  IMG_1251.JPG  IMG_1252.JPG  IMG_1253.JPG  IMG_1254.JPG |
| 10034 | Village: Ramchandra, Ward:08, Union: Dohilpara, Sadar, Chapainawabganj | IMG_1255.JPG  IMG_1256.JPG  IMG_1257.JPG  IMG_1258.JPG  IMG_1259.JPG  IMG_1260.JPG  IMG_1261.JPG  IMG_1262.JPG  IMG_1263.JPG  IMG_1264.JPG  IMG_1265.JPG  IMG_1266.JPG  IMG_1267.JPG  IMG_1268.JPG  IMG_1270.JPG  IMG_1271.JPG  IMG_1272.JPG  IMG_1273.JPG  IMG_1274.JPG  IMG_1275.JPG  IMG_1276.JPG  IMG_1277.JPG  IMG_1278.JPG  IMG_1279.JPG |
| 10035 | Village: Ramchandra, Ward:08, Union: Dohilpara, Sadar, Chapainawabganj | IMG_1280.JPG  IMG_1281.JPG  IMG_1282.JPG  IMG_1283.JPG  IMG_1284.JPG  IMG_1285.JPG  IMG_1286.JPG  IMG_1288.JPG  IMG_1289.JPG  IMG_1290.JPG  IMG_1291.JPG  IMG_1292.JPG |
| 10036 | Village: Ramchandra, Ward:08, Union: Dohilpara, Sadar, Chapainawabganj | IMG_1293.JPG  IMG_1294.JPG  IMG_1295.JPG  IMG_1296.JPG  IMG_1297.JPG  IMG_1298.JPG  IMG_1299.JPG  IMG_1300.JPG  IMG_1301.JPG  IMG_1302.JPG  IMG_1303.JPG  IMG_1304.JPG  IMG_1305.JPG  IMG_1306.JPG  IMG_1307.JPG  IMG_1308.JPG  IMG_1309.JPG  IMG_1310.JPG  IMG_1311.JPG  IMG_1312.JPG  IMG_1313.JPG |
| 10037 | Village: Ramchandra, Ward:08, Union: Dohilpara, Sadar, Chapainawabganj | IMG_1314.JPG  IMG_1315.JPG  IMG_1316.JPG  IMG_1317.JPG  IMG_1318.JPG  IMG_1319.JPG  IMG_1320.JPG  IMG_1321.JPG  IMG_1322.JPG  IMG_1323.JPG  IMG_1324.JPG  IMG_1325.JPG  IMG_1326.JPG  IMG_1327.JPG  IMG_1328.JPG  IMG_1329.JPG  IMG_1330.JPG  IMG_1331.JPG  IMG_1332.JPG  IMG_1333.JPG  IMG_1334.JPG  IMG_1335.JPG  IMG_1336.JPG  IMG_1337.JPG  IMG_1338.JPG  IMG_1339.JPG  IMG_1340.JPG |

## Image Information of Healthy Subjects Having Resemblance to Arsenic-Affected Skin:

Among the 741 images of healthy subjects, some images contain resemblances to arsenic-affected skin. This information may help some interested researchers in further research. The list is provided below:

| - 1. IMG_1968.JPG   2. IMG_1969.JPG   3. IMG_1970.JPG   4. IMG_1971.JPG   5. IMG_1972.JPG   6. IMG_1973.JPG   7. IMG_1974.JPG   8. IMG_1975.JPG   9. IMG_1976.JPG   10. IMG_1977.JPG   11. IMG_1978.JPG   12. IMG_1979.JPG   13. IMG_1980.JPG   14. IMG_1981.JPG   15. IMG_1982.JPG   16. IMG_1983.JPG   17. IMG_1984.JPG   18. IMG_1985.JPG   19. IMG_1986.JPG   20. IMG_1987.JPG   21. IMG_1988.JPG   22. IMG_1989.JPG   23. IMG_1990.JPG   24. IMG_1991.JPG   25. IMG_1992.JPG   26. IMG_1993.JPG   27. IMG_1994.JPG   28. IMG_1995.JPG   29. IMG_1996.JPG   30. IMG_1997.JPG   31. IMG_1998.JPG   32. IMG_1999.JPG   33. IMG_2001.JPG   34. IMG_2002.JPG   35. IMG_2003.JPG   36. IMG_2004.JPG   37. IMG_2005.JPG   38. IMG_2006.JPG   39. IMG_2007.JPG   40. IMG_2008.JPG   41. IMG_2009.JPG   42. IMG_2010.JPG   43. IMG_2011.JPG   44. IMG_2012.JPG   45. IMG_2013.JPG   46. IMG_2014.JPG   47. IMG_2015.JPG   48. IMG_2016.JPG   49. IMG_2017.JPG   50. IMG_2018.JPG   51. IMG_2019.JPG   52. IMG_2024.JPG | - 1. IMG_2020.JPG   2. IMG_2021.JPG   3. IMG_2022.JPG   4. IMG_2023.JPG   5. IMG_2025.JPG   6. IMG_2026.JPG   7. IMG_2027.JPG   8. IMG_2028.JPG   9. IMG_2029.JPG   10. IMG_2030.JPG   11. IMG_2031.JPG   12. IMG_2032.JPG   13. IMG_2033.JPG   14. IMG_2034.JPG   15. IMG_2036.JPG   16. IMG_2037.JPG   17. IMG_2038.JPG   18. IMG_2039.JPG   19. IMG_2040.JPG   20. IMG_2041.JPG   21. IMG_2042.JPG   22. IMG_2043.JPG   23. IMG_2044.JPG   24. IMG_2045.JPG   25. IMG_2046.JPG   26. IMG_2047.JPG   27. IMG_2048.JPG   28. IMG_2049.JPG   29. IMG_2057.JPG   30. IMG_2058.JPG   31. IMG_2059.JPG   32. IMG_2060.JPG   33. IMG_2061.JPG   34. IMG_2062.JPG   35. IMG_2063.JPG   36. IMG_2064.JPG   37. IMG_2065.JPG   38. IMG_2066.JPG   39. IMG_2067.JPG   40. IMG_2068.JPG   41. IMG_2069.JPG   42. IMG_2070.JPG   43. IMG_2071.JPG   44. IMG_2072.JPG   45. IMG_2073.JPG   46. IMG_2091.JPG   47. IMG_2092.JPG   48. IMG_2093.JPG   49. IMG_2094.JPG   50. IMG_2095.JPG   51. IMG_2096.JPG   52. IMG_2097.JPG |
| --- | --- |

The provided dataset intends to provide visual information about some specific areas of Bangladesh. Hopefully, this dataset will be beneficial to researchers who are interested in working in dermatology and enhance practical use with the help of computer vision.

-------
